# Advanced Treatment of Coking Wastewater by Polyaluminum Silicate Sulfate for Organic Compounds Removal

**DOI:** 10.3390/ijerph20146342

**Published:** 2023-07-11

**Authors:** Jiangnan Wang, Fang Chang, Maosheng Zheng

**Affiliations:** 1 Marine Resources Research Centre, Tianjin Research Institute for Water Transport Engineering, Ministry of Transport, Tianjin 300456, China; 2 Key Laboratory of Resources and Environmental Systems Optimization, Ministry of Education, College of Environmental Science and Engineering, North China Electric Power University, Beijing 102206, China; maoshengzheng@ncepu.edu.cn

**Keywords:** coking wastewater, polyaluminum silicate sulfate, PASS, coagulation, advanced treatment

## Abstract

Coking wastewater is a typical high-strength organic wastewater, for which it is difficult to meet discharging standards with a single biological treatment. In this study, effective advanced treatment of coking wastewater was achieved by coagulation with freshly prepared polyaluminum silicate sulfate (PASS). The performance advantage was determined through comparison with commercial coagulants including ferric chloride, polyferric sulfate, aluminum sulfate and polyaluminum chloride. Both single-factor and Taguchi experiments were conducted to determine the optimal conditions for coagulation with COD_Cr_ and UV_254_ as indicators. A dosage of 7 mmol/L PASS, flocculation velocity of 75 r/min, flocculation time of 30 min, pH of 7, and temperature of 20 °C could decrease the COD_Cr_ concentration from 196.67 mg/L to 59.94 mg/L. Enhanced coagulation could further help to remove the organic compounds, including pre-oxidation with ozonation, adsorption with activated carbon, assistant coagulation with polyacrylamide and secondary coagulation. UV spectrum scanning and gas chromatography-mass spectrometry revealed that the coagulation process effectively removed the majority of organic compounds, especially the high molecular weight alkanes and heterocyclic compounds. Coagulation with PASS provides an effective alternative for the advanced treatment of coking wastewater.

## 1. Introduction

Coking wastewater is a high-strength industrial wastewater generated from coal coking, coal gas purification, and by-product recovery processes of the coking industry [1]. It usually contains dozens of inorganic pollutants (ammonium, sulfate, cyanide, thiocyanate, etc.) and organic contaminants, such as phenolic compounds, polycyclic aromatic hydrocarbons (PAHs), nitrogen- and sulfur-containing heterocyclic compounds, etc., [2]. Most of these constituents are persistent, toxic, mutative, and carcinogenic, which can cause respiratory diseases, reproductive diseases, genetic diseases and so on [3]. Therefore, coking wastewater poses a severe threat to the environment and human health. Especially in China, where coal is a major energy source, hundred million tons of coking wastewater (1.8 × 10^8^ t/a) is generated each year [4]. Therefore, efficient treatment of coking wastewater has become an urgent problem.

Conventional coking wastewater treatment procedures include pretreatment and biological treatment [5,6]. Activated sludge process is the most frequently adopted biological treatment, which can effectively remove ammonia and short-chain fatty acids [7]. However, it is difficult for refractory organics such as PAHs and heterocyclic compounds to achieve a high removal efficiency, although some new biological methods have been developed and employed, including powdered activated carbon treatment (PACT) process, photosynthetic bacteria method, combined hydrolytic acidification, and aerobic treatment [2,7,8,9,10]. As a result, it is difficult to meet the discharge requirements using conventional coking wastewater treatment, hence post-treatment becomes a focus for current researchers [11]. Common advanced treatment methods include catalytic ozonation, active carbon adsorption, ion exchange and reverse osmosis, which usually require a high-cost investment and operation [12,13,14,15,16].

As an advanced treatment method, coagulation is advantageous due to its merits of low cost and easy operation [17]. Aluminum and ferric salts are the most frequently used coagulants, which are efficient in the removal of color, suspended soils and some organic compounds in the wastewater industry [18,19,20]. The coagulation process efficiently removes these pollutants by decreasing or neutralizing the electric charge or zeta potential, which facilitates the aggregation of colloidal and fine suspended materials to form microflocs [21,22]. Polymeric flocculants could further bring together the microflocs to form large agglomerations through physical mixing and binding actions [23,24]. However, the traditional aluminum and ferric coagulants still have the disadvantages of large dosage and dissatisfactory performance, especially when treating industrial wastewater for organic compound removal [25,26]. A relatively new inorganic polymer coagulant, polyaluminum silicate sulfate (PASS), was developed in the 1990s and has attracted wide attention as the introduction of silicate components significantly improved the contaminants removal capability, ascribed to the large floc size, strong adsorption bridging and wide pH adaption ability [27,28,29].

This research aims to: (i) prepare a polymeric coagulant polyaluminum silicate sulfate and to determine its effectiveness in the advanced treatment of coking wastewater through comparison with commercial aluminum and ferric chloride; (ii) to further evaluate the optimal conditions of removing organic compounds represented by COD_Cr_ and UV_254_ by single factor and Taguchi experiments; (iii) to finally explore the removal mechanisms through UV scanning and GC-MS detection of the wastewater before and after treatment by polyaluminum silicate sulfate.

## 2. Materials and Methods

### 2.1. Wastewater

The raw coking wastewater used for the coagulation tests was collected from the effluent of a lab-scale biological anaerobic and aerobic filter treating coking wastewater, which was originally taken from a coking plant in Tangshan, Hebei province. The average COD_Cr_ and UV_254_ value of the effluent was 196.67 mg/L and 3.358 cm^−1^, respectively. The temperature and pH averaged at 20 °C and 8.32, respectively. Notably, only the removal of organic compounds was emphasized, as the ammonium had been degraded to as low as 5.12 mg/L by the biological process [30,31].

### 2.2. Preparation of PASS Coagulants

The polyaluminum silicate sulfate (PASS) used in this study was prepared in the laboratory using the modified method with an Al/Si ratio of 5 [32]. In addition, commercial coagulants including ferric chloride (FC), polyferric sulfate (PFS), aluminum sulfate (AS), and polyaluminum chloride (PAC) were also employed and conducted in the coagulation experiments parallelly with PASS.

### 2.3. Jar Tests

The jar tests were carried out in a six-paddle agitation apparatus to determine the coagulation performance at varied dosage and conditions. Firstly, 150 mL coking wastewater in a beaker was mixed with the coagulant at a high speed (230 r/min) for 30 s, followed by a slow mixing (75 r/min) for 15 min. After settling for 30 min, the supernatant was sampled for determining the COD_Cr_ and UV_254_ values. The dosage of PASS and the commercial coagulants was expressed by the molar concentration (in mmol/L) of active component (aluminum or ferrum) [33]. At the optimal dosage of PASS, single-factor experiment at varied flocculation intensity, flocculation time, settling time, pH value and temperature were conducted to determine the optimal coagulation conditions with COD_Cr_ and UV_254_ values as the main indicators [34,35]. Taguchi L16 experiment at four levels of varied factors was further conducted to explore the optimized coagulation conditions.

### 2.4. Enhanced Coagulation Experiment

A series of enhanced coagulation experiments were conducted to explore the potential enhancement on the coagulation efficiencies, such as pre-oxidation, active carbon adsorption, assisted coagulation by polyacrylamide, and secondary coagulation. In the pre-oxidation, KMnO_4_, NaClO, O_3_ was used as the oxidizer and reacted for 20 min prior to the jar-test coagulation. In the adsorption, activated carbon at a dosage of 0~500 mg/L was added to the coking wastewater prior to the PASS addition. In the assisted coagulation, polyacrylamide dosed at 0~5 mg/L was supplemented to the reaction system at the beginning of the slow mixing step. In the secondary coagulation, the collected supernatant after coagulation was implemented to a repetitive coagulation with PASS dosage at 0~8 mmol/L. When each enhanced coagulation experiment was completed after settling for 30 min, the supernatant was sampled and COD_Cr_ and UV_254_ values were determined.

### 2.5. Analytical Methods

The supernatant was filtrated with a 0.45 μm filter membrane before analysis. COD_Cr_ concentration and UV_254_ were determined by the potassium dichromate oxidation method and UV absorption value at 254 nm, respectively. The scan of the wastewater was conducted by UV–vis spectrophotometer with wavelengths ranging from 190~600 nm.

Gas chromatography-mass spectrometry (GCMS) analysis was carried out to analyze the removal performance of organic compounds by the coagulation. Briefly, 200 mL coking wastewater in a 500 mL separating funnel was extracted three times with 10 mL dichloromethane at neutral, alkaline and acidic conditions, respectively. The extracted liquid was condensed into 1 mL with high-purity nitrogen gas and 2 μL was injected to analysis by 6890 N/5973 GCMS system (Agilent Corporation, Santa Clara, CA, USA) with a modified condition referring to a previous study [33]. A BP-5MS capillary column with an inner diameter of 0.20 mm and a length of 12 m was used in the separation system with high-purity helium gas as the carrier gas. Inlet temperature was maintained at 280 °C. The temperature control program included an initial temperature of 40 °C for 15 min, followed by increasing to 280 °C with an increment of 10 °C/min and maintaining for 5 min. Electron ionization with the electron energy and electron multiplier voltage at 70 eV and 1200 V, respectively. Full scan mode ranged from 50 to 500 amu. The organic compounds peaking at different retention times were identified through matching their mass spectrum with the standard mass spectral library NIST 11.

## 3. Results and Discussion

### 3.1. Coagulation Performance of PASS

**Figure 1:**
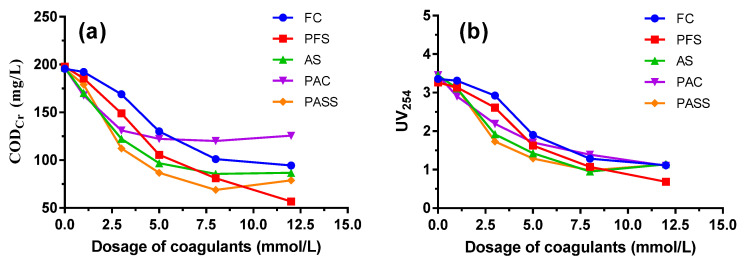
COD_Cr_ (**a**) and UV_254_ (**b**) removal performance by polyaluminum silicate sulfate (PASS) and commercial coagulants including ferric chloride (FC), polyferric sulfate (PFS), aluminum sulfate (AS), and polyaluminum chloride (PAC).

As can be seen from Figure 1, COD_Cr_ and UV_254_ values obviously decreased with increasing coagulant dosage in the beginning, and the trend slowed down later on. An increasing tendency even emerged as coagulant dosage increased to higher than 8 mmol/L. At a small dosage of coagulants, the removal efficiency of organic compounds was relatively low due to the difficulty of flocs formation or sedimentation of small flocs. With the dosage increasing, flocs grow bigger and the precipitation also gets faster, which speeds up the removal efficiency. At a low dosage of coagulants, the main coagulation mechanisms are charge neutralization and adsorption bridging, while at high dosage adsorption bridging as well as net capturing and precipitation play a vital role in organics removal [21,36].

Compared to the commercial coagulants, PASS showed the best coagulation performance at a dosage of 3~8 mmol/L. It could be seen that when the dosage of PASS was 5 mmol/L, the COD_Cr_ value in the discharged water reached 86.67 mg/L, meeting the standard of the Integrated Wastewater Discharge Standard of China (GB 8978-1996). PASS also achieved the best removal efficiency of UV_254_ at the dosage of 3~5 mmol/L. As UV_254_ could represent the content of high weight molecular organic compounds containing C=C and C=O, such as humic and aromatic compounds, it is suitable to be used to reflect the concentration of refractory organics in coking wastewater, which usually correlates well to COD_Cr_ values. The dosage of 5 mmol/L was recommended to be the application dosage, taking financial costs into consideration, and was chosen as the optimal value for the following jar test in this study.

### 3.2. Optimal Factors Affecting Coagulation Performance

#### 3.2.1. Effect of Flocculation Speed and Time

**Figure 2:**
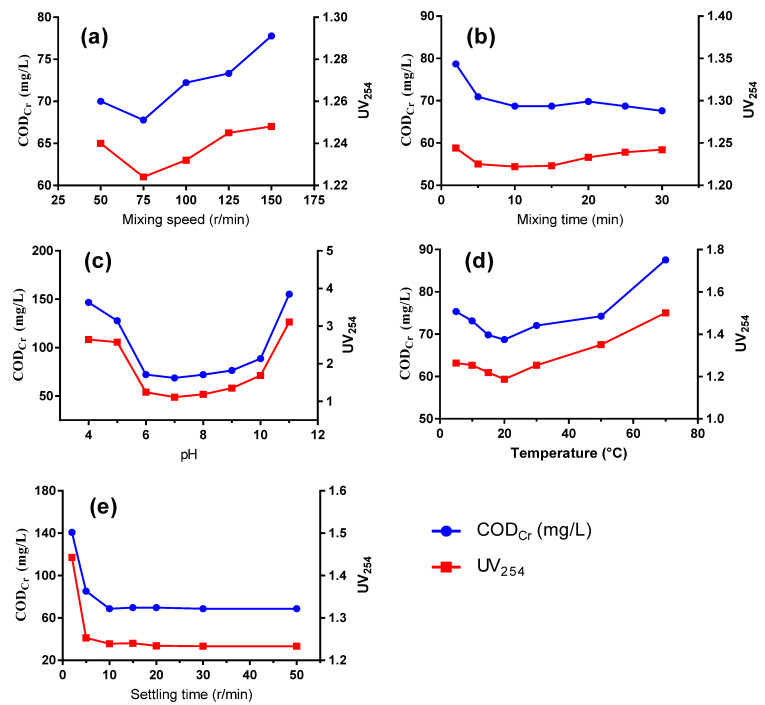
COD_Cr_ and UV_254_ removal performance by PASS at varied mixing speeds (**a**), mixing time (**b**), pH (**c**), temperature (**d**), and settling time (**e**).

As shown in Figure 2, operation conditions posed a significant impact on the removal of organic compounds, including mixing speed, mixing time, pH, temperature and settling time. As the mixing speed increased, the removal efficiency reached a high of 75 r/min and decreased at higher speeds (Figure 2a). Low flocculation speed would result in insufficient contact between flocs and organic compounds, while too high a speed would cause vigorous stirring and shear force that would not only hinder the formation of large flocs but also break newly formed flocs. Generally, the COD_Cr_ concentration decreases when extending flocculation time, as time is needed for floc formation and agglomeration. However, as time progressed longer than 10 min, the removal efficiency of COD_Cr_ and UV_254_ no longer exhibited a downward trend (Figure 2b).

#### 3.2.2. Effect of pH Value

Optimal coagulation performance could be realized under neutral conditions, while an acidic or alkaline condition is not suitable for the removal of organics (Figure 2c). When the pH increased to around 7, the hydrolysis of PASS was promoted with the production of Al(OH)_3_, which is an amphoteric compound that presents in different forms as the pH varies. Under neutral conditions, [Al(OH)_2_(H_2_O)_4_]^+^ and [AI(OH)_3_(H_2_O)_3_] are the primary forms, when the aluminum hydroxide flocs from the hydrolysis of aluminum-based compounds reach the highest concentration, thereby performing well the roles of compressing the double electric layers and charge neutralization. The zero-potential point of PASS was pH 5.0. Under neutral conditions, the hydrolyzed polysilicate sol-gel performed well at adsorption bridging and the net trapping effect, effectively achieving contaminant removal efficiency together with Al(OH)_3_. Moreover, the ζ potential of the organics in wastewater approaches 0 under neutral or weak acidic conditions, leading to a decreasing solubility as the number of organics in the ionic state reduces while that in the molecular state increases [18]. Therefore, more organics would be absorbed into the abundant aluminum hydroxide flocs and be efficiently removed after co-precipitation.

#### 3.2.3. Impact of Temperature

As can be seen from Figure 2d, the optimal water temperature was 20 °C, while a higher or a lower temperature impedes the removal efficiency of the organic compounds. At a low temperature, the water viscosity increases, which results in weaker Brownian movement and weaker anisotropic flocculation. Moreover, the efficient hydrolysis of the coagulant is inhibited, which poses an impact on the destabilization of colloidal particles and hinders the adsorption process. Too high a temperature causes petty flocs and makes precipitation difficult due to the increased kinetic energy of molecular motion. In addition, a high temperature causes high moisture content of the sludge, increasing the disposal cost.

#### 3.2.4. Impact of Settling Time

As depicted in Figure 2e, COD_Cr_ and UV_254_ values decreased more sharply with the increase of sedimentation time. Nonetheless, the measured performance remained relatively stable after 10 min when a clear sludge-water interface formed and the residual suspended particles in the supernatant would hardly settle. Finally, a relatively longer time of 30 min was chosen for optimal sedimentation to ensure a dense sludge and a stable removal efficiency.

### 3.3. Optimal Results by Taguchi Experiments

**Figure 3:**
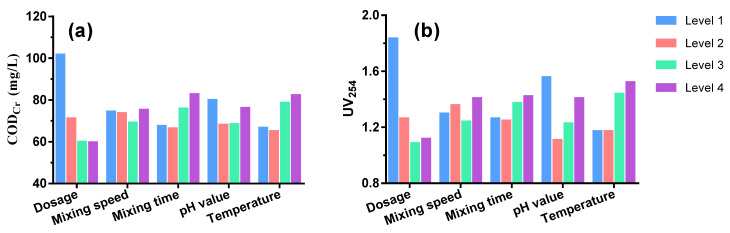
Taguchi L16 experiment results of COD_Cr_ (**a**) and UV_254_ (**b**) removal performance by PASS at four levels of varied factors.

The optimal conditions were determined by a Taguchi L16 experiment and range analysis (Table 1). The order of impact on COD_Cr_ removal efficiency was as follows: dosage > temperature > flocculation time > pH value > mixing speed. The optimal condition for COD_Cr_ removal included a coagulant dosage of 9 mmol/L, temperature of 20 °C, flocculation time of 10 min, pH value of 7 and mixing speed of 90 r/min (Figure 3). For UV_254_ removal efficiency, the order of impact could be seen as follows: dosage > pH > temperature > flocculation time > mixing speed. The optimal conditions were similar to those for COD_Cr_ except for a temperature of 15 °C. Taking into consideration both COD_Cr_ and UV_254_, the optimal conditions were chosen as: coagulant dosage of 7 mmol/L, mixing speed of 90 r/min, flocculation time of 10 min, pH value of 7, and temperature of 20 °C. Under these conditions, COD_Cr_ could be reduced to as low as 59.94 mg/L, meeting the standard of make-up water in industrial recirculating cooling systems (≤60 mg/L) as required by the Code for Design of Industrial Recirculating Cooling Water Treatment (GB/T 50050-2017).

### 3.4. Enhanced Coagulation Results

#### 3.4.1. Pre-Oxidation

**Figure 4:**
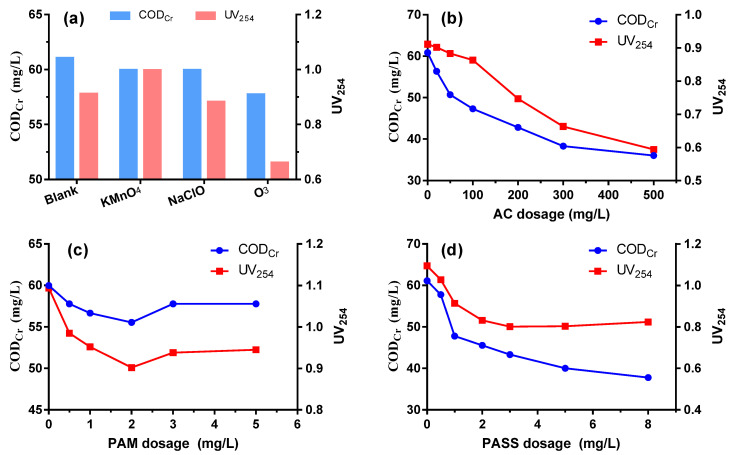
COD_Cr_ and UV_254_ removal performance enhanced by pre-oxidation (**a**), adsorption (**b**), flocculation aid (**c**) and secondary coagulation (**d**).

The potential enhancement of the coagulation performance with the help of other physico-chemical methods was investigated, which is of significance especially for wastewater that is notoriously difficult to purify by a single coagulation process. As shown in Figure 4a, compared to the blank, pre-oxidation by KMnO_4_ and NaClO did not realize any improvement of COD_Cr_ removal; KMnO_4_ even contributes to some UV_254_ increase due to its own colority. Only pre-oxidation by ozone had obvious benefit to the removal of organic compounds, especially in achieving a high UV_254_ removal due to its efficient decoloration performance.

#### 3.4.2. Activated Carbon Adsorption

A significant enhancement of COD_Cr_ and UV_254_ removal was realized by synergetic active carbon adsorption. COD_Cr_ could be reduced to 50.70 mg/L at a dosage of 50 mg/L and further decreased with the increase of activated carbon addition (Figure 4b). On one hand, the gigantic surface of activated carbon powder has a strong absorption for dissolved organics, especially low molecular weight compounds, forming a complementation effect with coagulation [37]. On the other hand, the activated carbon powder could provide flocculation centers, which is essential for flocs formation and is beneficial for the removal efficiency of particle colloidal substances.

#### 3.4.3. Coagulant Aid Polyacrylamide

As is depicted in Figure 4c, assisted coagulant PAM is effective in augmenting the decrease of COD_cr_ and UV_254_, and its optimal dosage is 2 mg/L. PAM’s polymerization degree ranges from 20,000 to 90,000, with a molecular weight between 1,500,000 and 6,000,000. Its strong adsorption for other solids’ surfaces and its bridging between colloidal particles help to promote the coagulation process. When the PAM dosage is relatively low, both adsorption and bridging effects are insignificant. However, when the PAM dosage is too high, itself would dissolve into the water, which increases the COD_Cr_ value. PAM could also greatly help to increase the sedimentation velocity of flocs, thus thickening the floc sediments [38].

#### 3.4.4. Secondary Coagulation

As can be seen from Figure 4d, secondary coagulation of the discharged water from coagulation could further enhance its removal efficiency of organics. With a dosage of 1 mmol/L OPASS, COD_Cr_ and UV_254_ dropped to 47.77 mg/L and 0.914, respectively. The COD_Cr_ and UV_254_ removal performance further improved as the dosage was increased. However, the financial costs for the infrastructure construction and management would definitely be increased when secondary coagulation is taken into consideration.

### 3.5. Coagulation Mechanism Exploration

#### 3.5.1. Ultraviolet Spectrum Scanning

**Figure 5:**
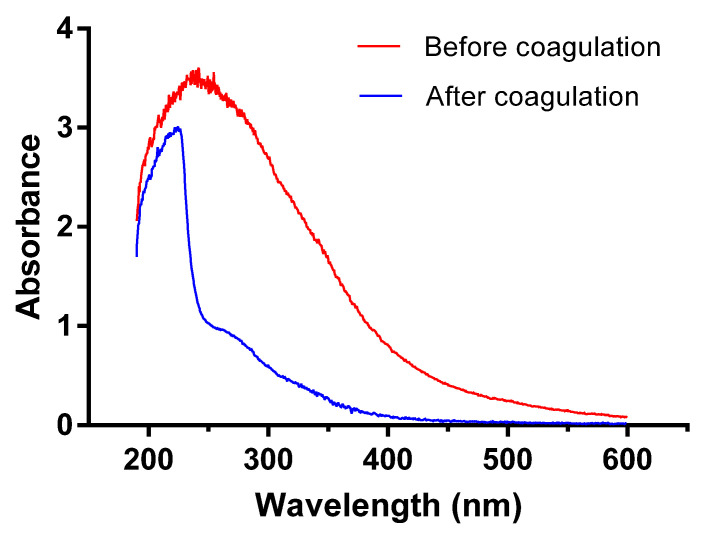
Ultraviolet spectroscopy scanning results.

According to spectrum analysis, saturated organic compounds exhibit no adsorption in near ultraviolet band while organics with conjugated double bonds or benzene rings have obvious adsorption in ultraviolet bands ranging from 200 to 400 nm. Specifically, the aromatic compounds with benzene rings adsorb spectrums mainly within 250–260 nm; polycyclic aromatic hydrocarbons (PAHs) exhibit strong adsorption near long wave ultraviolet bands [33]. Thus, ultraviolet spectroscopy scanning could offer clues about the relative concentrations of these organic compounds. As can be seen from Figure 5, ultraviolet spectrum absorbance is relatively lower in discharged wastewater after coagulation than that before treatment, especially in UV spectrums between 250–400 nm. High absorbance of raw water between 200–300 nm suggests a large amount of compounds with conjugated double bonds (such as conjugated diene, unsaturated aldehyde, and unsaturated ketone), or aromatic compounds (such as arene, polyromantic hydrocarbon, and heterocyclic aromatic compounds). Discharged water after coagulation exhibits strong UV absorption between 200–250 nm, suggesting that the coagulation process is not ideal in removing compounds with conjugated double bonds; on the contrary, a sharp decrease in UV adsorption between 250–300 nm indicates the efficient elimination of aromatic compounds.

#### 3.5.2. Gas Chromatography-Mass Spectrometry (GC-MS)

**Figure 6:**
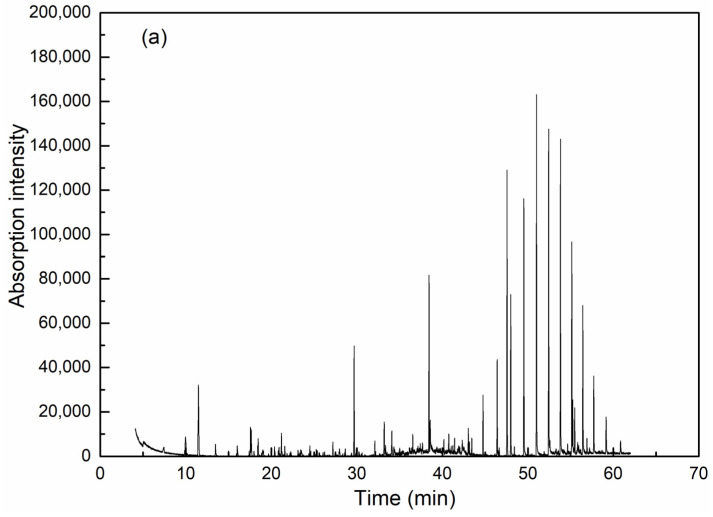

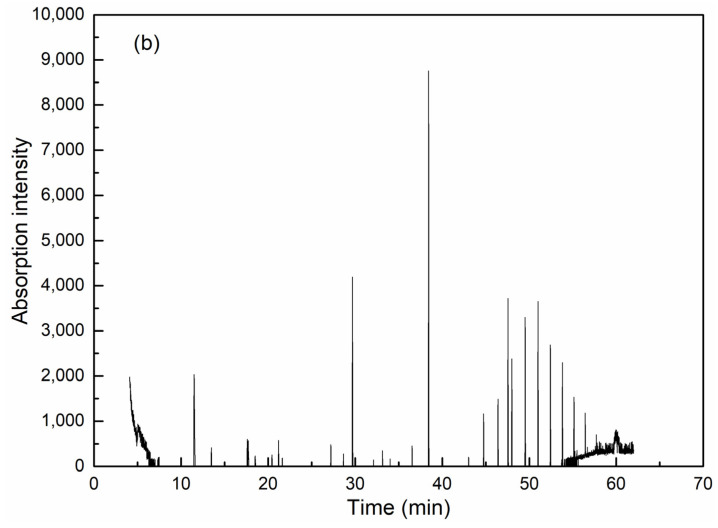
GC-MS analysis results for wastewater before (**a**) and after (**b**) coagulation.

GC-MS is effective for detection and analysis in water quality testing, especially in the isolation, identification and quantification of complicated organic components. As can be seen from Figure 6 and Table 2, coagulation is obviously effective in removing organic compounds. Among the extractable organic compounds by dichloromethane, 19 types of organics with a matching degree over 70 and relative concentration over 0.5% could be detected in raw wastewater, among which there were six kinds of alkane, five heterocyclic compounds, one olefin, one acid, one ester and one phenolic compound. High molecular weight alkanes take up the highest concentration. After coagulation, only six kinds of organics were detected (including two kinds of alkane, one heterocyclic compound, one ester and one phenolic compound) and all have an identical peaking time with the corresponding compounds in raw water, which thereby could be assumed as the exact species from raw water before coagulation.

As the concentration of organic compounds was very low in the wastewater after coagulation, the precise GC-MS detection of some trace organics could not be guaranteed. However, considering that the extraction and analytical procedures were the same for both wastewaters before and after treatment, the removal performances of certain organic compounds could still be illustrated qualitatively within the detection range of GC-MS. It could be seen that water after coagulation mainly consists of high molecular weight alkane compounds, mostly heneicosane, however, the removal rate reached as high as 98%. The amount of esters ranks the second in discharged water due to its high stability in water, although only 1,2-benzenedicarboxylic acid, butyl cyclohexyl ester was left with the removal efficiency of 91.08%. For 2-cyclohexen-1-one with the shortest retention time, the removal efficiency reached 94.13%, although the small hydrophilic molecular organic was difficult to capture by coagulants [34]. For heterocyclic compounds, only 1,8-naphthalimide could be detected in the discharged wastewater, indicating the efficient removal of the coagulation. In contrast to the traditional aluminum, the introduction of silica into the hydrolyzing metal promoted the increase of molecular size and enhanced the bridge-aggregating ability to capture and sediment the contaminants in water [25]. Therefore, the removal of organic compounds, especially high molecular weight alkane and heterocyclic compounds was well achieved by PASS. Considering the hazard to the natural water body and to human beings for high toxicity and resistance to biodegradation, coagulation with PASS as an advanced treatment of coking wastewater provides an alternative way to solve this problem.

## 4. Conclusions

The coagulant polyaluminum silicate sulfate (PASS) was prepared in this study and applied in the advanced treatment of coking wastewater, which showed the best coagulation performance compared with commercial coagulants. At a dosage of 5 mmol/L, the COD_Cr_ value after treatment reduced to 86.67 mg/L from 196.67 mg/L. Both single-factor and Taguchi experiments revealed that the optimal conditions for the removal of organics include: dosage of 7 mmol/L, flocculation velocity of 75 r/min, flocculation time of 30 min, pH of 7, and temperature of 20 °C. Under these optimal conditions, the COD_Cr_ concentration could be further decreased to 59.94 mg/L. Enhanced coagulation experiments such as pre-oxidation with ozonation, active carbon adsorption, PAM assistance and secondary coagulation could further reduce the COD_Cr_ concentration. Ultraviolet spectrum scanning and GC-MS analysis suggest that the coagulation process with PASS played a vital role in the removal of organic compounds, especially for high molecular weight alkane and heterocyclic compounds, providing an alternative for the advanced treatment of coking wastewater.

## Figures and Tables

**Table 1:** Taguchi L16 experiment design and the corresponding results.

	Dosage (mmol/L)	Mixing Speed (r/min)	Mixing Time (min)	pH Value	Temperature (°C)	COD_Cr_ (mg/L)	UV254
1	3	50	5	6	15	97.68	1.518
2	3	70	10	7	20	82.14	1.416
3	3	90	15	8	25	101.01	1.810
4	3	110	20	9	30	125.43	2.289
5	5	50	10	8	30	69.93	1.252
6	5	70	5	9	25	74.37	1.426
7	5	90	20	6	20	75.48	1.348
8	5	110	15	7	15	64.38	1.019
9	7	50	15	9	20	58.83	1.030
10	7	70	20	8	15	58.83	0.959
11	7	90	5	7	30	54.39	0.915
12	7	110	10	6	25	67.71	1.433
13	9	50	20	7	25	71.04	1.078
14	9	70	15	6	30	78.81	1.623
15	9	90	10	9	15	45.51	0.880
16	9	110	5	8	20	43.29	0.884

**Table 2:** GC-MS analysis of organic compounds in raw and discharged wastewater.

Retention Time (min)	Organic Compound Name	Before Coagulation		After Coagulation		Removal Efficiency (%)
		Peak Area	Proportion (%)	Peak Area	Proportion (%)	
11.479	2-cyclohexen-1-one	1,620,727	3.09	95,208	12.30	94.13
17.555	1-ethyl-4-piperidone	344,708	0.66			
17.646	3-ethyl-2,4-dimethylpyrrole	416,969	0.79			
21.181	benzothiazole	264,600	0.50			
29.675	1,8-naphthalimide	1,338,682	2.55	98,566	12.73	92.64
33.200	cyclopentyl-hydantoin	562,414	1.07			
38.441	1,2-benzenedicarboxylic acid, butyl cyclohexyl ester	2,135,102	4.07	190,546	24.61	91.08
38.575	hexadecanoic acid	845,638	1.61			
40.752	methylcholanthrene	458,356	0.87			
43.030	tetratriacontane	286,472	0.55			
44.753	docosane	670,453	1.28			
46.405	docosane	1,138,506	2.17			
47.561	2,4-bis(1-phenylethyl) phenol	3,352,312	6.39	83,596	10.80	97.51
47.999	heneicosane	1,966,420	3.75	47,928	6.19	97.56
49.529	heneicosane	3,264,155	6.22	69,020	8.91	97.89
51.010	heneicosane	4,398,514	8.38	79,435	10.26	98.19
52.433	heneicosane	4,159,765	7.92	60,745	7.84	98.54
52.578	dqualene	424,800	0.81			
53.813	pentatriacontane	4,063,962	7.74	49,293	6.37	98.79
55.140	octacosane	2,695,909	5.14			
56.429	heptacosane	2,080,623	3.96			
57.707	henicosane	1,053,509	2.01			
59.151	tetratriacontane	636,520	1.21			

## Data Availability

Not applicable.
